# Comparative morphology of immature stages of four species of *Chinavia* (Hemiptera, Pentatomidae), with a key to the species of Rio Grande do Sul, Brazil

**DOI:** 10.3897/zookeys.319.4310

**Published:** 2013-07-30

**Authors:** Brenda Bianca Rodrigues Jesse Fürstenau, Cristiano Feldens Schwertner, Jocelia Grazia

**Affiliations:** 1 Department of Zoology, Universidade Federal do Rio Grande do Sul (UFRGS), Av. Bento Gonçalves, 9500. Prédio 43435, sala 216 – 91501-970 Porto Alegre, Rio Grande do Sul, Brazil; 2 Department of Biological Sciences, Universidade Federal de São Paulo (UNIFESP), Campus Diadema, Rua Prof. Artur Riedel, 275 – 09972-270 Eldorado, Diadema, São Paulo, Brazil

**Keywords:** Hemiptera, Heteroptera, Pentatomidae, nymphs, stink bugs, keys, pest, Brazil, Neotropical Region

## Abstract

*Chinavia* Orian (1965) is one of the most diverse genera of Pentatomidae, distributed in the Afrotropical, Neotropical and Nearctic Regions. Thirty-two species are recorded for Brazil, some of them having potential economic impact because they are found on crops and referred to as pests. The morphology of the five nymphal instars of *Chinavia armigera* (Stål, 1859), *Chinavia aseada* (Rolston, 1983), *Chinavia brasicola* (Rolston, 1983) and *Chinavia runaspis* (Dallas, 1851) are described here. Through a comparative study, identification keys were developed to allow an early identification of the 12 *Chinavia* species of Rio Grande do Sul.

## Introduction

The majority of the morphological and taxonomic studies of stink bugs concerns only the adult forms. However, because of taxonomic, economic and ecological importance, the study of the immature forms has been increased in the last years. When associated with crops of economic importance, an oportune identification of the species allows an early adoption of pest control, reducing production losses (Brailovsky et al. 1992). Also, the description of the morphology of stink bug eggs and nymphs can contribute with new characters for classification and phylogenies (Matesco et al. 2009b). The knowledge of immature stages also enables more reliable estimation of the richness and abundance of species and helps to assess the impact of environmental changes (Costa et al. 2006; Mendonça et al. 2009).

The nymphal stage in Pentatomidae is divided into five instars, which can be distinguished by body size, general coloration, presence and distribution of maculae, and degree of development of scutellum and pterothecae in the first and second thoracic segments (DeCoursey and Esselbaugh 1962; Jones and Coppel 1963; Brailovsky et al. 1992). The nymphal stages of Pentatomidae have characters that allow the construction of identification keys (DeCoursey and Allen 1968; Saini 1989) to recognize the instars or the families (Southwood 1956; Leston and Scudder 1956; Costa et al. 2006); these keys often give more emphasis to characteristics of the fifth instar.

The genus *Chinavia* was proposed in 1965 by Orian; it was treated as a subgenus of *Acrosternum* Fieber, 1860. On the basis of morphological evidence and in agreement with the proposals of Day (1965), Roche (1977) and Ahmad (1996), Schwertner and Grazia (2006) treated the taxon as a full genus. Currently, there are 84 species described for the genus, distributed in the Afrotropical, Nearctic and Neotropical regions (Schwertner and Grazia 2006). In Brazil, 32 species are recorded, 18 of which are endemic (Schwertner and Grazia 2007). Twelve species can be found in the state of Rio Grande do Sul, eight of which have the morphology of nymphs or some biological aspects known: *Chinavia erythrocnemis* (Berg, 1878) (Matesco et al. 2006), *Chinavia impicticornis* (Stål, 1872) (Grazia et al. 1982), *Chinavia longicorialis* (Breddin, 1901) (Matesco et al. 2009a), *Chinavia musiva* (Berg, 1878) (Matesco et al. 2008), *Chinavia nigridorsata* (Breddin, 1901) (Vecchio et al. 1988), *Chinavia obstinata* (Stål, 1860) (Matesco et al. 2003), *Chinavia pengue* (Rolston, 1983)(Matesco et al. 2007) and *Chinavia ubica* (Rolston, 1983) (Schwertner et al. 2002).

This paper describes the morphology of the five nymphal instars of *Chinavia armigera* (Stål, 1859), *Chinavia aseada* (Rolston, 1983), *Chinavia brasicola* (Rolston, 1983) and *Chinavia runaspis* (Dallas, 1851). The morphology of the eggs of these four species will be described and discussed in a future paper. Identification keys for each instar are also provided.

## Material and methods

Adults and nymphs were collected in four locations in the state of Rio Grande do Sul, Brazil, between March 2007 and March 2011 (Table 1). Individuals were reared in the laboratory under controlled conditions (24 ± 1°C; 70 ± 10% RH and photoperiod of 12 h). Eggs and first instar nymphs were kept in Petri dishes with a moistened cotton pad. From the second instar to adulthood, the insects were reared in 500-ml plastic pots covered with organdy. To maintain the humidity inside the pot, an Eppendorf tube containing water, covered with cotton, was used.

**Table 1.** Collecting sites of four *Chinavia* species.

**Species**	**Collecting site in Brazil**	**Geographical coordinates**
*Chinavia armigera* (Stal)	Rio Grande, RS	32.5719S, 52.5593W
*Chinavia aseada* (Rolston)	Passo Fundo, RS	28.1546S, 53.2424W
*Chinavia brasicola* (Rolston)	São Francisco de Paula, RS	29.4239S, 50.3872W
*Chinavia runaspis* (Dallas)	Porto Alegre, RS	30.0693S, 51.2422W

Green beans (*Phaseolus vulgaris* L.) (Fabaceae) and fruits of cherry tomatoes (*Lycopersicon esculentum* Mill.) (Solanaceae) were offered as food. Individuals of *Chinavia aseada* and *Chinavia brasicola* were also offered soybean (*Glycine max* L.) and peanut (*Arachis hypogaea* L.) (Fabaceae), respectively. The food was replaced twice a week. During both nymphal and adult stages, the pots were replaced by cleaned ones whenever necessary.

The color of the nymphs was observed in live individuals. The specimens selected for analysis of morphology were kept frozen and were fixed in 70% alcohol after completion of the study.

Terminology for eggs follows Matesco et al. (2006), and for nymphs Davidová-Vilímová and Podoubský (1999) and Matesco et al. (2009b). Measurements are in millimeters corresponding to mean ± SD, obtained according Matesco et al. (2009b). Photographs were obtained with a digital camera (Sony DSC-HX1 or Nikon Coolpix 995) attached to a stereomicroscope. Drawings were made with a camera lucida coupled to a stereomicroscope, digitally scanned and edited with Adobe Illustrator® and Adobe Photoshop®. Voucher specimens were deposited at the Entomological Collection of the Department of Zoology, Federal University of Rio Grande do Sul (DZRS).

## Results

### Chinavia armigera

(Stål, 1859)

http://species-id.net/wiki/Chinavia_armigera

Figs 1–6

#### Remarks.

Adults of *Chinavia armigera* have a general body color green to dark green, with a median longitudinal line and margins of the body yellowish (Rolston 1983). The distribution includes Brazil (Rio Grande do Sul), Argentina and Uruguay (Schwertner and Grazia 2007). *Chinavia armigera* is associated with plants such as yerba mate *(Ilex paraguariensis* St. Hil) (Aquifoliaceae), soybean (*Glycine max* L.) (Fabaceae), sugarcane (*Saccharum* L.) (Poaceae), cotton (*Gossypium hirsutum* L.) (Malvaceae) and rice (*Oryza sativa* L.) (Poaceae). The morphometric parameters of nymphal instars are shown in Table 2.

**Table 2.** Morphometric traits of nymphs of *Chinavia armigera* (Stål, 1859) (n = 15) (mean ± standard deviation, mm).

**Measures**	**1st instar**	**2nd instar**	**3rd instar**	**4th instar**	**5th instar**
TL	2.02±0.09	3.39±0.26	4.69±0.31	6.23±0.15	8.23±1.05
TW	1.49±0.10	2.45±0.27	3.95±0.40	4.27±0.34	9.52±4.21
ID	0.62±0.15	0.75±0.06	1.1±0.08	1.21±0.11	1.54±0.13
I	0.10±0.08	0.15±0.05	0.2±0.09	0.29±0.09	0.38±0.11
II	0.20±0.08	0.43±0.09	0.72±0.05	1.2±0.08	1.27±0.24
III	0.17±0.09	0.36±0.05	0.62±0.08	0.85±0.15	1.34±0.29
IV	0.39±0.09	0.47±0.12	0.93±0.09	1.12±0.19	1.33±0.09
RL	0.79±0.17	1.44±0.16	1.51±0.3	2.32±0.32	3.01±0.31
PL	0.24±0.08	0.59±0.10	0.98±0.33	1.17±0.22	2±0.12
PW	0.31±0.04	0.53±0.09	1.01±0.33	1.13±0.33	1.89±0.32

HL head length; ID interocular distance; PL pronotum length; PW pronotum width; RL rostrum length; TL total length; TW total width; I, II, III, IV length of antennal segments.

#### First instar

(Fig. 2). Body round and convex, surface without punctuation. General color dark brown to black. Head conical and strongly declivent; black, with median orange macula, which extends from the posterior portion of the head to the posterior margin of metanotum; clypeus with apex obtuse, surpassing mandibular plates, these subtriangular shaped. Ocelli absent. Antennae black, intersegmental areas with light brown color; antennal segments with short hairs well distributed. Antennal segment I shortest and antennal segment IV longest; antennal segments II and III subequal. Rostrum black, reaching the metacoxae. Thorax mostly dark, except for the median orange macula. Legs black, with hairs uniformly distributed on all segments, tibiae ventrally cylindrical and dorsally flattened, tarsi with two segments, a pair of tarsal claws and pulvili. Abdomen dark brown to black with the three pairs (3+3) of white maculae located between lateral dorsal plates and the first three median dorsal plates, and a white rounded median macula located anteriad of the first median dorsal plate. Dorso-abdominal scent glands ostioles of anterior, median and posterior glands present on dorsal plates placed at intersegmental line between the abdominal terga 3-4, 4-5 and 5-6 respectively. Dorsal median and lateral plates black, without punctuation, the lateral semi-circular, adjacent to the lateral margin of each segment. Spiracles near anterior margin of lateral plates, on abdominal segments II to VIII. From urosternites III to VII, 1+1 trichobothria placed medially of an imaginary line across spiracles and near posterior margin of each segment.

#### Second instar

(Fig. 3). Body oval, less convex than in the first instar. Head predominantly black colored, densely punctured on the dorsum. Clypeus with apex obtuse, subequal to mandibular plates, larger than in the previous instar. Eye diameter equal to width of clypeus at base. Rostrum black, not reaching metacoxae. Thorax with 1+1 orange maculae along the margins of the pronotum; margins of pronotum and mesonotum serrate and slightly deflected. Hairs more abundant on the tibiae. Abdomen mostly dark brown; dorsal abdominal maculae distributed as follows: 1+1 white, rounded, located between the lateral and the first dorsal median plates; a white macula, small, rounded, located anteriad of first median dorsal plate; 4+4 white maculae between the lateral and median dorsal plates. Dorsal median and lateral plates black and punctured. From urosternites III to VII, 2+2 trichobothria, one trichobothrium placed medially of the spiracular line and the other along that line. Other characteristics as described for the first instar.

#### Third instar

(Fig. 4). Mandibular plates subtriangular, length subequal to clypeus, whose apex is obtuse. Thorax densely punctured, predominantly black, except for the orange maculae on anterolateral margins, which are finely crenulated. Abdomen with a pair (1+1) of white, large and rounded maculae, one on each side of the first dorsal median plate; small white macula anteriad of first dorsal median plate; four pairs (4+4) of white maculae between the lateral and median dorsal plates. Black median and lateral dorsal plates, strongly punctured. Lateral plates semicircular in shape, with orange macula in the center, emarginated in black. Other characteristics as described in previous instars.

#### Fourth instar

(Fig. 5). Body oval, predominantly dark brown. Mandibular plates with broad bands and clypeus with a thin median strip straw-yellow. Rostrum black, not reaching metacoxae. Thorax predominantly black, except a straw-yellow macula without defined shape and an orange macula along the anterolateral margins. Posterior margin of mesonotum sinuous, denoting the scutellum and formation of wing pads, which reach the posterior margin of metanotum. Abdomen dark brown, sparse punctuation, not as dense as on the thorax, abdominal maculae distributed as follows: 1+1 white, round, large, located between the lateral plates and the first median dorsal plate; a white and oval macula located anteriad of the first median dorsal plate; 4+4 white maculae located between the dorsal median plates and the dorsal lateral plates; 1+1 white, small, rounded macula located along the posterolateral margins of the second median dorsal plate. Lateral plates semicircular, orange emarginated in black, slightly punctured; median plates predominantly black, coarsely punctured, with sparse brownish maculae. Other characteristics as described in previous instars.

#### Fifth instar

(Fig. 6). Body oval to pyriform. Head flat, punctured. Mandibular plates predominantly straw-yellow, with black border and orange band extending from the anterior margin of the eyes to the apex of the clypeus, which is black, with a straw-yellow median strip, wider in the posterior portion, on the edge of the pronotum. Antennae generally straw-yellow, with black maculae. Thorax predominantly straw-yellow, with dark brown and sparse maculae, shapeless, producing a variegated appearance. Margins of pronotum and mesonotum serrate, with a pair (1+1) of anterolateral orange maculae. Pronotum wider; mesonotum more developed; scutellum well delimited. Wing pads well developed, surpassing the middle of abdominal segment III. Legs straw-yellow, with dark margins; hairs more abundant on the tibiae. Abdomen predominantly brown, with the same distribution and number of maculae observed in the 4th instar. Margin of the median dorsal plates darker than the center. Median dorsal plates predominantly orange. Urosternite VIII split longitudinally in females and entire in males. Other characteristics as described in previous instars.

**Figures 1–6.**
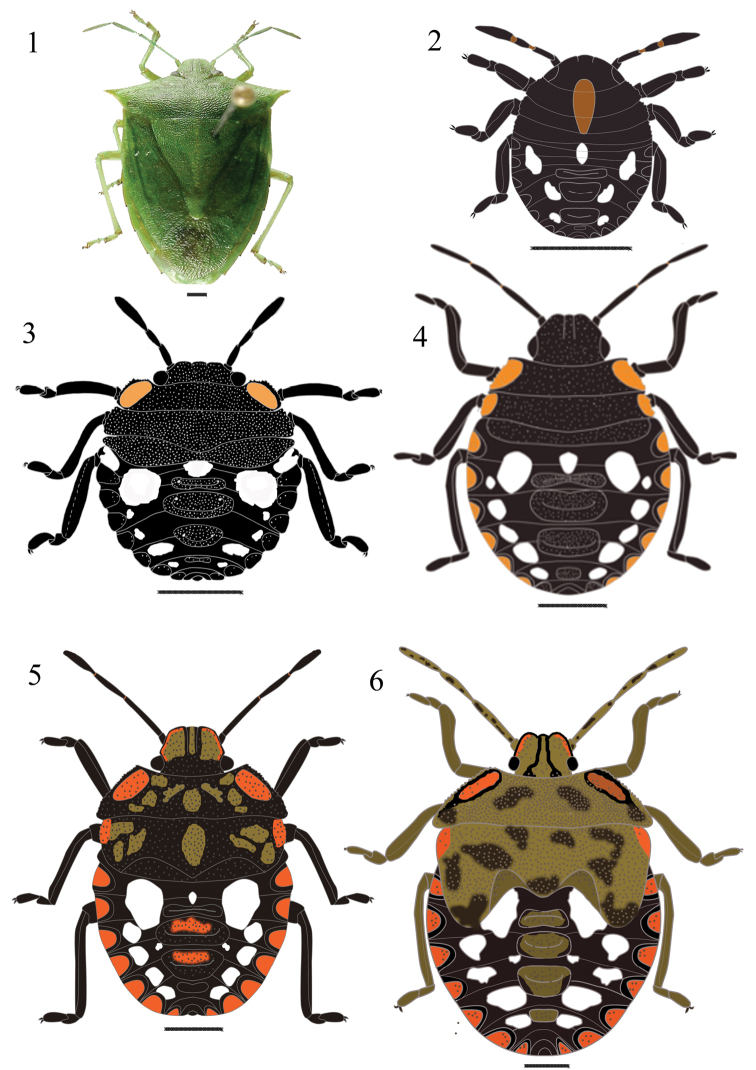
*Chinavia armigera* (Stål, 1859). **1** Adult **2** First instar **3** Second instar **4** Third instar **5** Fourth instar **6** Fifth instar.

### Chinavia aseada

(Rolston, 1983)

http://species-id.net/wiki/Chinavia_aseada

Figs 7–12

#### Remarks.

Adults of *Chinavia aseada* have general body color green and margins of the body, head, pronotum, hemelytra and connexivum reddish (Rolston 1983). Distribution includes Brazil (Pará, Mato Grosso, Distrito Federal, Paraná and Rio Grande do Sul) (Schwertner and Grazia 2007) and northern Argentina (Schwertner and Grazia 2007). The morphometric parameters of nymphal instars are shown in Table 3.

**Table 3.** Morphometric traits of nymphs of *Chinavia aseada* (Rolston, 1983) (n = 5) (mean ± standard deviation, mm).

**Measures**	**1st instar**	**2nd instar**	**3rd instar**	**4th instar**	**5th instar**
TL	2.03±0.42	3.01±0.33	3.97±0.31	5.98±0.45	9.34±0.89
TW	1.54±0.23	2.36±0.27	4.06±0.27	4.89±0.22	10.09±1.23
ID	0.63±0.03	0.85±0.06	1.1±0.08	1.32±0.14	1.59±0.17
I	0.11±0.05	0.16±0.04	0.21±0.06	0.33±0.05	0.41±0.11
II	0.22±0.03	0.42±0.05	0.72±0.06	1.1±0.08	1.32±0.14
III	0.23±0.05	0.41±0.05	0.62±0.07	0.92±0.12	1.39±0.21
IV	0.41±0.08	0.65±0.11	0.95±0.08	1.17±0.09	1.43±0.12
RL	0.7±0.17	1.36±0.14	1.55±0.34	2.47±0.33	3.78±0.31
PL	0.33±0.03	0.58±0.07	1.06±0.34	1.27±0.32	2.01±0.27
PW	1.23±0.08	2.17±0.14	3.14±0.29	4.32±0.31	5.17±0.29

HL head length; ID interocular distance; PL pronotum length; PW pronotum width; RL rostrum length; TL total length; TW total width; I, II, III, IV length of antennal segments.

#### First instar

(Fig. 8). Body round and convex, surface without punctuation. General color dark brown to black. Head conical and strongly declivent; black, with median orange macula, which extends from the posterior portion of the head to the posterior margin of metanotum; clypeus with apex obtuse, not surpassing mandibular plates, these subtriangular shaped. Ocelli absent. Antennae black, intersegmental areas with orange color; antennal segments with short and uniformly distributed hairs. Antennal segment I shortest and antennal segment IV longest. Antennal segments III and IV subequal in size. Rostrum black, surpassing metacoxae.Thorax mostly dark, except for the orange median macula. Legs black, with hairs uniformly distributed on all segments, tibiae dorsally flattened, tarsi with two segments, a pair of tarsal claws and pulvili. Abdomen predominantly black, with white maculae distributed as follows: one pair (1+1) round and large, located between the lateral plates and the first median dorsal plate; a small macula anteriad of first median dorsal plate; two pairs (2+2) of small, located along the margins of the lateroposterior margins of the second and third median dorsal plates. Median dorsal plates black; ostioles on median dorsal plates I-III. Lateral plates semicircular, black, without punctuation, adjacent to lateral margin of each segment. Spiracles near ventral anterior margin of lateral plates, on urosternites II to VIII. From urosternites III to VII, 1+1 trichobothria placed medially of an imaginary line across spiracles and near posterior margin of each segment.

#### Second instar

(Fig. 9). Body oval and less convex than first instar. Head less declivent than in previous instar, predominantly black, coarsely punctured on the dorsum. Clypeus obtuse at apex, subequal in size to the mandibular plates, larger than in previous instar. Eyes almost as wide as clypeus at base. Rostrum black, not reaching the metacoxae. Thorax with 1+1 orange maculae along the anterolateral margins of pronotum; lateral margins of pro- and mesonotum slightly deflected. Legs black, dense hairs on tibiae. Abdomen predominantly black, with 5+5 white maculae between lateral and median dorsal plates, one white small macula anteriad offirst median dorsal plate. Median and lateral dorsal plates black punctured. On the ventral plates, 2+2 trichobothria on urosternites III to VII. One trichobothrium medially of the spiracular line and the other along that line. Other characteristics as described for the first instar.

#### Third instar

(Fig. 10). Some specimens can have an orange band in the middle of each mandibular plate. Thorax densely punctured, predominantly black, except for a few orange spots, with irregular shape and size. Pronotum trapezoidal, with 1+1 orange maculae along the anterolateral margins, which are serrate. Margins of mesonotum slightly serrate, with a pair (1+1) of orange maculae. Abdomen with a pair (1+1) of white, large maculae, which has approximately circular shape, located on each side of the first median dorsal plate. Lateral plates semicircular, orange outlined in black. A white and small macula, located above first median dorsal plate and two pairs (2+2) of white maculae between lateral and median dorsal plates. Median dorsal plates predominantly black, densely punctured, with irregularly shaped orange maculae located in the middle of the plate. Other characteristics as described in the previous instars.

#### Fourth instar

(Fig. 11). Body oval, predominantly black. Head less declivent than in third instar, large light orange bands on mandibular plates, clypeus black. Antennae light brown. Maculae on the dorsum of the thorax shapeless, the same color as the bands on mandibular plates. Pronotum trapezoidal; mesonotum rectangular, posterior margin wide, “V” shaped, denoting the formation of scutellum. Wing pads slightly developed, reaching posterior margin of metanotum. Legs light brown with black borders. Rostrum black, reaching metacoxae. Abdomen light brown, punctuation sparse, not as dense as on the thorax; white maculae distributed as follows: one pair (1+1) of round-shaped, large, located between lateral and median plates; one rounded, located anteriad of first median dorsal plate; four pairs (4+4) of white maculae, located between lateral and median dorsal plates. Lateral plates semicircular, orange outlined in black, slightly punctured; second and third median dorsal plates predominantly black, coarsely punctured with sparse orange maculae located in the middle of the plates. Other characteristics as described in the previous instars.

#### Fifth instar

(Fig. 12)**.** Body oval to pyriform. Head flat, slightly punctured. Eyes with transverse straw-yellow band. Mandibular plates predominantly straw-yellow, with black margins. Clypeus predominantly black, with median straw-yellow band, wider in the posterior portion. Antennae predominantly straw-yellow. Thorax predominantly straw-yellow to light brown, with dark brown maculae, sparse, shapeless, producing a variegated appearance. Lateral margins of pronotum and mesonotum depressed, slightly serrate and deflected. Pronotum wide; mesonotum more developed; scutellum well delimited. Wing pads well developed, surpassing the middle of abdominal segment III. Legs straw-yellow to dark brown, with dark margins; hairs more abundant on the tibiae. Abdomen predominantly light brown, with same number and distribution of maculae observed in fourth instar. Median plates predominantly orange. Urosternite VIII split longitudinally in females and entire in males. Other characteristics as described in the previous instars.

**Figures 7–12.**
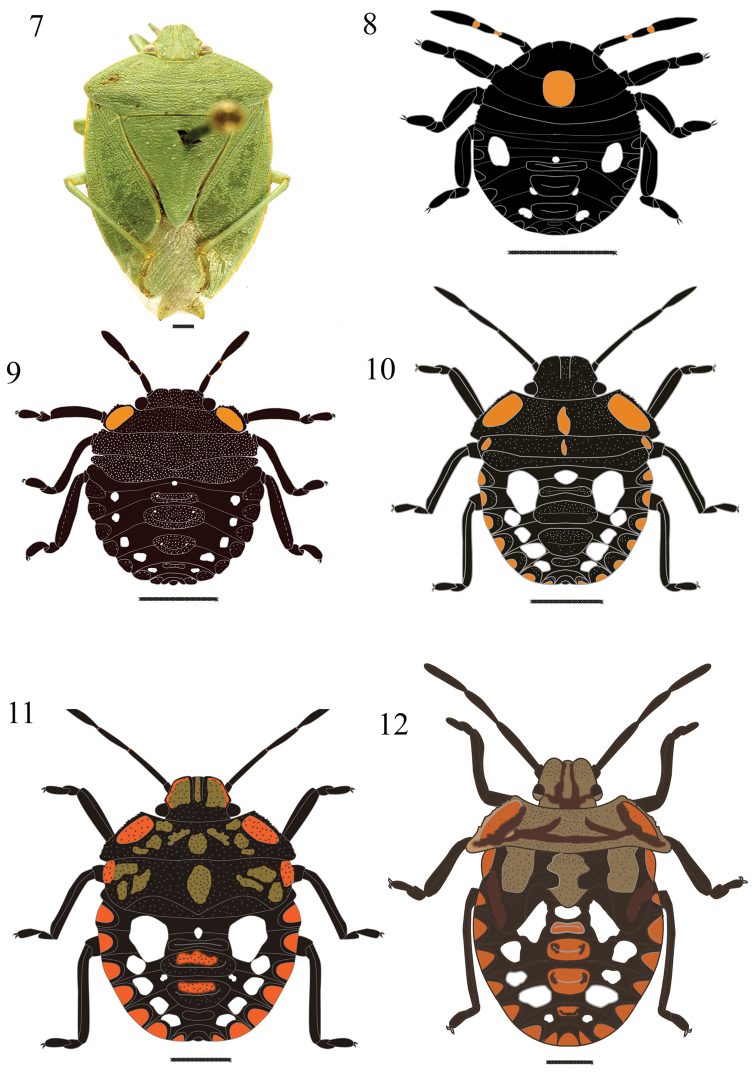
*Chinavia aseada* (Rolston, 1983). **7** Adult **8** First instar **9** Second instar **10** Third instar **11** Fourth instar **12** Fifth instar.

### Chinavia brasicola

(Rolston, 1983)

http://species-id.net/wiki/Chinavia_brasicola

Figs 13–18

#### Remarks.

Adults have general body color green, connexivum red-orange with black maculae on posterolateral angles (Rolston 1983). The distribution includes the Brazilian states of São Paulo, Santa Catarina and Rio Grande do Sul (Schwertner and Grazia 2007). The single sitting record is a fern *Dennstaedtia globulifera* (Poir.) Hieron (Pteridophyta) (Schwertner and Grazia 2007). The morphometric parameters of nymphal instars are shown in Table 4.

**Table 4.** Morphometric traits of nymphs of *Chinavia brasicola* (Rolston, 1983) (n = 15) (mean ± standard deviation, mm).

**Measures**	**1st instar**	**2nd instar**	**3rd instar**	**4th instar**	**5th instar**
TL	2.11±0.38	3.5±0.25	4.83±0.33	6.89±0.56	10.34±0.78
TW	1.69±0.04	2.68±0.25	4.04±0.26	5.24±0.34	11.52±17.23
ID	0.65±0.06	0.89±0.06	1.3±0.08	1.46±0.11	1.72±0.17
I	0.13±0.04	0.18±0.05	0.2±0.05	0.36±0.08	0.42±0.12
II	0.23±0.06	0.47±0.09	0.79±0.05	1.2±0.08	1.67±0.14
III	0.23±0.04	0.39±0.05	0.64±0.08	0.96±0.11	1.44±0.22
IV	0.44±0.09	0.67±0.12	0.92±0.09	1.22±0.09	1.63±0.12
RL	0.8±0.17	1.46±0.16	1.57±0.3	2.67±0.32	3.98±0.41
PL	0.32±0.04	0.63±0.09	1.02±0.33	1.26±0.22	2±0.2
PW	1.31±0.08	2.29±0.14	3.21±0.29	4.47±0.31	6.32±0.35

HL head length; ID interocular distance; PL pronotum length; PW pronotum width; RL rostrum length; TL total length; TW total width; I, II, III, IV length of antennal segments.

#### First instar

(Fig. 14). Body round and convex surface without punctuation. General coloration dark brown to black. Head conical and strongly declivent; black, with median orange macula, which extends from the posterior portion of the head to the anterior margin of mesonotum; clypeus with apex obtuse, slightly surpassing the mandibular plates, these subtriangular shaped. Ocelli absent. Antennae black; intersegmental areas with orange color; antennal segments with short and uniformly distributed hairs. Antennal segment I shortest and antennal segment IV longest. Antennal segments III and IV subequal in size. Rostrum black, reaching anterior margin of urosternite II. Thorax mostly dark, except for the orange median macula. Legs black, with hairs uniformly distributed on all segments, tibiae dorsally flattened, tarsi with two segments, a pair of tarsal claws and pulvili. Abdomen predominantly black, with three pairs (3+3) of white maculae, located between lateral and median plates. Median dorsal plates black; ostioles on median dorsal plates I-III. Lateral plates semicircular, black, without punctuation, adjacent to lateral margin of each segment. Spiracles near anterior margin of lateral plates, on urosternites II to VIII. From urosternites III to VII, 1+1 trichobothria placed medially of an imaginary line across spiracles and near posterior margin of each segment.

#### Second instar

(Fig. 15). Body oval and less convex than first instar. General color black, with punctuation on the dorsum of the head, thorax, median and lateral plates. Head less declivent than in previous instar. Clypeus obtuse at apex, subequal in size to the mandibular plates, which are broader than that observed in previous instar. Eyes almost as wide as base of clypeus. Rostrum black, surpassing the anterior margin of urosternite III. Thorax with 1+1 orange maculae along the margins of pronotum; lateral margins of pro- and mesonotum slightly deflected. Legs black, hairs more dense on tibiae. Abdomen predominantly black, maculae distributed as follows: one, white, round maculae, located anteriad of first median plate; a pair (1+1) of white maculae, located on each side of the first lateral plates; a pair (1+1) of large, rounded, orange maculae, located between lateral plates and first median plate and four pairs (4+4) white, located between lateral and median plates, the third pair is the largest. Median and lateral dorsal plates black punctured. Urosternites III to VII with 2+2 trichobothria, one trichobothrium medially of the spiracular line and the other along that line. Other characteristics as described for the first instar.

#### Third instar

(Fig. 16). Antennal segment I shorter, segments II and IV subequal in length, larger than segment III. Thorax punctured, predominantly black, except an orange maculae along each margin. Pronotum with margins slightly deflected and serrate. Legs black, except for the area between the femur and tibia, which has light brown color; hairs denser on the ventral surface of tibia and tarsus. Abdomen with a round white macula anteriad of first median plate; one pair (1+1) of orange maculae between the first median plate and lateral plates, and four pairs (4+4) of white maculae located between median and lateral plates of tergites IV-VII. Other characters as described for the previous instar.

#### Fourth instar

(Fig. 17). Body oval, less convex than in earlier instars, predominantly black. Head less declivent than in previous instar, black, with punctures. Some specimens with an orange band in the middle of each mandibular plates. Antennae with abundant hairs on segments III and IV. Thorax predominantly black, with orange maculae at margins of pro- and mesonotum. Pronotum trapezoidal; mesonotum rectangular, posterior margin wide, V-shaped, denoting the formation of scutellum. Wing pads slightly developed, reaching posterior margin of metanotum. Dorsal abdominal maculae with the same color and distribution as observed in third instar, but wider. Lateral plates semicircular, predominantly orange, slightly punctured. Other characteristics as described in the previous instars.

#### Fifth instar

(Fig. 18). Body oval to pyriform, predominantly black. Head flat; mandibular plates wide, each with orange band present in some individuals. Thorax predominantly black, 1+1 orange maculae on pronotum and mesonotum along the anterolateral margins, median macula orange. Pronotum wider; mesonotum more developed; scutellum well delimited. Wing pads well developed, surpassing the middle of abdominal segment III. Legs black, hairs denser ventrally. Abdomen black, coarsely punctured with maculae dorsally distributed as follows: one pair (1+1) of orange maculae near first median plate, with another white, semicircular macula between the preceding two maculae; and four pairs (4+4) of white maculae, located near lateral plates, on segments IV to VII; lateral plates with semicircular orange maculae, outlined in black. Urosternite VIII split longitudinally in females and entire in males. Other characteristics as described in the previous instars.

**Figures 13–18.**
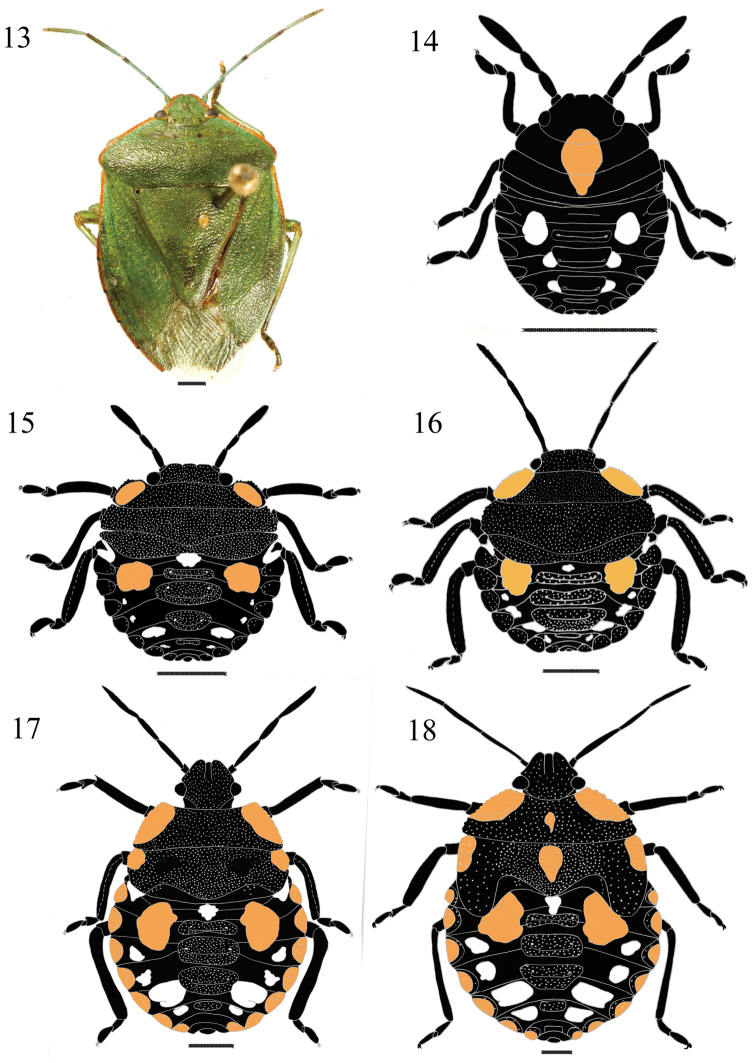
*Chinavia brasicola* (Rolston, 1983). **13** Adult **14** First instar **15** Second instar **16** Third instar **17** Fourth instar **18** Fifth instar.

### Chinavia runaspis

(Dallas, 1851)

http://species-id.net/wiki/Chinavia_runaspis

Figs 19–24

#### Remarks.

Adults of *Chinavia runaspis* havegeneral body color light green to dark green, with reddish orange color on the margins of mandibular plates, pronotum, basal third of hemelytra and connexivum (Schwertner and Grazia 2007). *Chinavia runaspis* is recorded for Venezuela, Suriname, Colombia, Brazil (Amapá, Pará, Minas Gerais, Rio de Janeiro, São Paulo, Santa Catarina, Rio Grande do Sul), Peru, Paraguay and Argentina (Schwertner and Grazia 2007). The morphometric parameters of nymphal instars are shown in Table 5.

**Table 5.** Morphometric traits of nymphs of *Chinavia runaspis* (Dallas, 1851) (n = 15) (mean ± standard deviation, mm).

**Measures**	**1st instar**	**2nd instar**	**3rd instar**	**4th instar**	**5th instar**
TL	2.35±0.44	3.7±0.31	4.9±0.35	8.1±1.21	11.31±1.21
TW	1.72±0.07	3.1±0.18	4.3±0.36	6.1±0.45	9.53±2.12
ID	0.7±0.05	0.92±0.07	1.6±0.12	1.88±0.32	1.92±0.17
I	0.14±0.06	0.23±0.08	0.27±0.05	0.4±0.23	0.43±0.03
II	0.24±0.02	0.49±0.08	0.78±0.09	1.4±0.34	1.72±0.23
III	0.23±0.04	0.4±0.05	0.77±0.07	1.3±0.08	1.62±0.22
IV	0.43±0.05	0.71±0.09	0.96±0.11	0.98±0.08	1.67±0.14
RL	0.9±0.09	1.39±0.05	1.7±0.8	0.96±0.11	3.44±0.22
PL	0.38±0.09	0.66±0.11	1.4±0.44	2.22±0.09	2.1±0.52
PW	1.4±0.33	2.01±0.16	3.9±0.39	5.1±1.1	6.5±1.2

HL, head length; ID, interocular distance; PL, pronotum length; PW, pronotum width; RL, rostrum length; TL, total length; TW, total width; I, II, III, IV, length of antennal segments.

#### First instar

(Fig. 20). Body round and convex, surface without punctuation. General coloration dark brown to black. Head conical and strongly declivent. Clypeus with apex obtuse, length subequal to that of mandibular plates, these subtriangular shaped. Ocelli absent. Diameter at eyes smaller than the base of clypeus. Antennae black, intersegmental areas light brown; antennal segments covered by few hairs sparsely distributed. Antennal segment I shortest and antennal segment IV longest. Antennal segments III and IV subequal in size. Rostrum black, slightly surpassing the mesocoxae. Thorax predominantly dark brown, with a large, rounded,orange macula, which extends from middle of the head to the posterior margin of mesonotum. Legs black, denser hairs on tibiae and tarsus; tarsi two-segmented with a pair of claws and pulvili. Tibiae dorsally flattened. Abdomen dark brown, with three pairs (3+3) of white maculae, located between lateral and median plates, which are black. Median dorsal plates black; ostioles on median dorsal plates I-III. Lateral plates semicircular, black, without punctuation, adjacent to lateral margin of each segment. Spiracles near anterior margin of lateral plates, on urosternites II to VIII. From urosternites III to VII, 1+1 trichobothria placed medially of an imaginary line across spiracles and near posterior margin of each segment.

#### Second instar

(Fig. 21). Body oval and less convex than in first instar. Head less declivent than previous instar, predominantly black, coarsely punctured in the dorsum. Clypeus obtuse at apex, subequal in size to the mandibular plates, which are broader than in previous instar. Eyes almost as wide as base of clypeus. Rostrum black, reaching the metacoxae. Thorax with 1+1 orange maculae along the margins of pronotum; lateral margins of pro- and mesonotum slightly deflected. Legs black, dense hairs on tibiae and tarsus. Abdomen predominantly dark brown, maculae distributed as follows: one pair (1+1) of orange, rounded and large maculae, and two pairs (2+2) of white and rounded maculae. Median and lateral dorsal plates black punctured. Urosternites III to VII with 2+2 trichobothria, one trichobothrium medially of the spiracular line and the other along that line. Other characteristics as described for the first instar.

#### Third instar

(Fig. 22). Mandibular plates and clypeus subequal in length. Thorax predominantly black, coarsely punctured, with a pair (2+2) of orange maculae along anterolateral margins, which are depressed, slightly deflected and not serrate. Abdomen predominantly black, with maculae distributed as follows: a white small, round macula, anteriad of first median plate; one pair (1+1) of large, orange, semicircular, located between first median and lateral plates; five pairs (5+5) of white and round maculae, located between lateral and median plates; and two pairs (2+2) of white, small maculae located near second and third median plates. All median and lateral plates predominantly black and coarsely punctured; lateral plates semicircular. Other characteristics as described in the previous instars.

#### Fourth instar

(Fig. 23). Body oval, less convex than in previous instars, predominantly black. Head less declivent than in third instar, clypeus black. Thorax predominantly black, except for shapeless, light orange maculae, on dorsum and margins. Maculae on the dorsum of the thorax light orange. Pronotum trapezoidal; mesonotum rectangular, posterior margin wide, V-shaped, denoting the formation of scutellum. Wing pads slightly developed, reaching posterior margin of metanotum. Lateral plates orange with black margin, with few punctures. Maculae on abdominal dorsum with the same coloration and distribution as in previous instar, but larger. Other characteristics as described in the previous instars.

#### Fifth instar

(Fig. 24). Body oval to pyriform. Head flat, slightly punctured; mandibular plates wide, each a longitudinal orange band. Thorax predominantly black with a pair (1+1) of orange maculae on pronotum along anterolateral margins and laterally on mesonotum. In some individuals, additional orange maculae are dispersed on the dorsum of the thorax. Pronotum wider, anterolateral margins slightly convex; mesonotum more developed, scutellum well delimited. Wing pads well developed, surpassing the middle of abdominal segment III. Legs black, hairs more abundant ventrally. Abdomen predominantly black, densely dotted, with maculae distributed as follows: one white macula anteriad of the first median plate; one pair (1+1) of orange maculae between lateral and first median plates; four pairs (4+4) white maculae lon segments IV-VII. Lateral plates semicircular, orange with black margins, with few punctures. Median plates predominantly black, coarsely punctured, with orange maculae in the center of the plate. Urosternite VIII split longitudinally in females and entire in males. Other characteristics as described in the previous instars.

**Figures 19–24.**
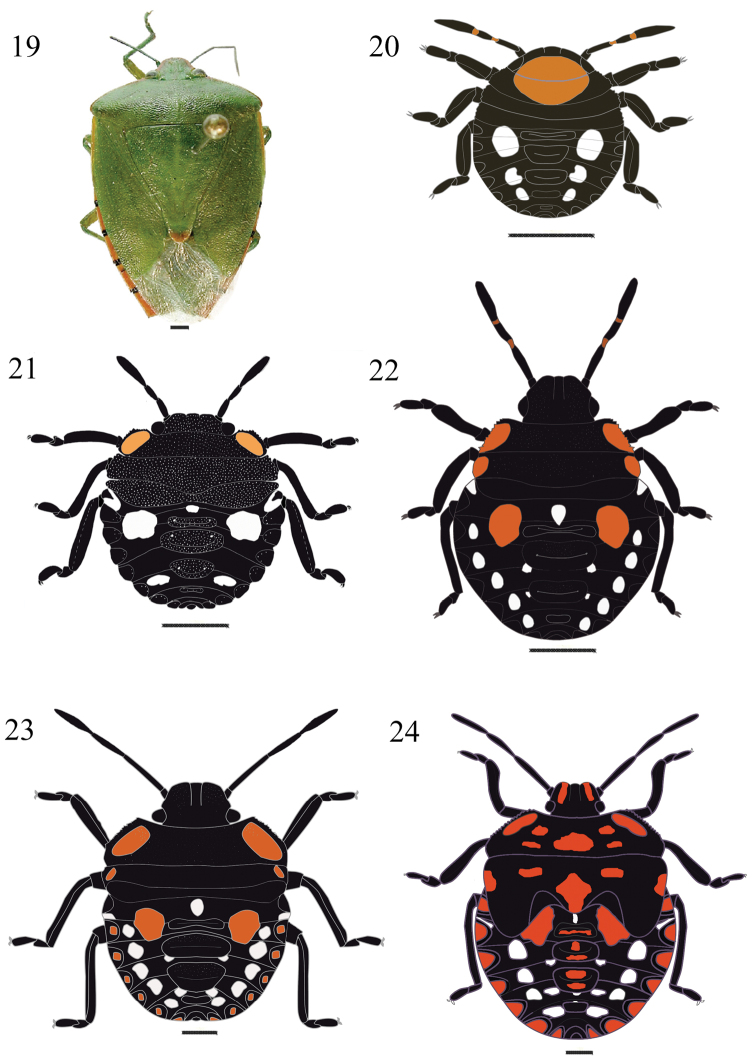
*Chinavia runaspis* (Dallas, 1851). **19** Adult **20** First instar **21** Second instar **22** Third instar **23** Fourth instar **24** Fifth instar.

##### Key to identification of first instars of *Chinavia* in Rio Grande do Sul.

**Table:** 

1	Body color predominantly brown to black; median dorsal maculae along head and thoracic segments	2
–	Body color predominantly green, variegated; median dorsal macula restricted to head	*Chinavia musiva* (Berg, 1878)
2	Head and legs entirely with the same predominant color of the body	3
–	Apex of head and areas of legs in a different color of the body coloration	*Chinavia erythrocnemis* (Berg, 1878)
3	Median dorsal maculae orange or reddish orange	4
–	Median dorsal maculae yellow	5
4	Abdominal dorsal maculae white	6
–	Abdominal dorsal maculae white and yellow to yellowish-orange	*Chinavia obstinata* (Stål, 1860)
5	Median macula oblong; macula anteriad to first median plate with twice the diameter of the eye	*Chinavia nigridorsata* (Breddin, 1901)
–	Median macula sub lozenge, macula anteriad to first median plate smaller than the diameter of the eye	*Chinavia impicticornis* (Stål, 1872)
6	Dorsal surface of abdomen with three pairs (3+3) of white maculae, located between lateral and median plates; white, small macula before first median plate absent	7
–	Dorsal surface of abdomen with white maculae arranged in a distinct pattern in addition to the white, small macula before first median plate	8
7	Median dorsal macula rounded, very large	*Chinavia runaspis* (Dallas, 1851)
–	Median dorsal macula almost oval, smaller	*Chinavia brasicola* (Rolston, 1983)
8	White macula anteriad of first median plate	9
–	White, rounded maculae before all median plates	*Chinavia ubica* (Rolston, 1983)
9	Thorax without maculae on lateral margins	10
–	Thorax with maculae on lateral margins	*Chinavia longicorialis* (Breddin, 1901)
10	Median dorsal macula almost rounded, extending from base of head to posterior margin of pronotum	*Chinavia aseada* (Rolston, 1983)
–	Median dorsal macula in a different shape to the above mentioned, covering base of head and surpassing the posterior margin of pronotum	11
11	Median dorsal macula reaching the posterior margin of metanotum, white maculae surrounding first lateral plate	*Chinavia pengue* (Rolston, 1983)
–	Median dorsal macula reaching the posterior margin of mesonotum, median plates without adjacent maculae	*Chinavia armigera* (Stål, 1859)

##### Key to identification of second instars of *Chinavia* in Rio Grande do Sul.

**Table:** 

1	Body color predominantly brown to black	2
–	Body color predominantly green, variegated	*Chinavia musiva* (Berg, 1878)
2	Apex of the head, and areas of antennae and legs red, thorax without maculae	*Chinavia erythrocnemis* (Berg, 1878)
–	Head, antennae and legs entirely with the same predominant color of the body; thoracic maculae in pronotum and /or mesonotum	3
3	Thoracic maculae yellow to orange	4
–	Thoracic maculae reddish	6
4	Thoracic maculae yellow	5
–	Thoracic maculae orange	7
5	First pair of lateral plates surrounded by white maculae, the remaining lateral plates with a white macula between them	*Chinavia nigridorsata* (Breddin, 1901)
–	First pair of lateral plates with a white macula near it; 5+5 white maculae between lateral and median plates	*Chinavia impicticornis* (Stål, 1872)
6	Body color predominantly brown, white maculae anteriad of median dorsal plates	*Chinavia ubica* (Rolston, 1983)
–	Body color predominantly black, one white macula anteriad of first median plate; 3+3 white maculae on abdomen	*Chinavia pengue* (Rolston, 1983)
7	Orange thoracic maculae on pronotal and mesonotal margins	*Chinavia longicorialis* (Breddin, 1901)
–	Orange thoracic maculae on pronotum margins only	8
8	Abdominal maculae white	9
–	Abdominal maculae in other colors	10
9	Abdomen with 5+5 small maculae	*Chinavia armigera* (Stål, 1859)
–	Abdomen with 1+1 large and 4+4 small maculae	*Chinavia aseada* (Rolston, 1983)
10	Abdomen with 1+1 yellow maculae between first median and lateral plates	*Chinavia obstinata* (Stål, 1860)
–	Abdomen with 1+1 orange maculae between first median and lateral plates	11
11	Abdomen with one white macula anteriad of first median plate; 1+1 orange maculae and 4+4 white maculae between median and lateral plates	*Chinavia brasicola* (Rolston, 1983)
–	Abdomen with 1+1 orange maculae and 1+1 white maculae posterior to the third median plate	*Chinavia runaspis* (Dallas, 1851)

##### Key to identification of third instars of *Chinavia* in Rio Grande do Sul.

**Table:** 

1	Body color predominantly brown to black	2
–	Body color predominantly green and variegated	*Chinavia musiva* (Berg, 1878)
2	Apex of the head, antenna segment I, apices of femora and bases of tibiae red	*Chinavia erythrocnemis* (Berg, 1878)
–	Head, antennae and legs with the same predominant color of the body	3
3	Median region of thorax without maculae; posterolateral angles of pronotum not produced	4
–	Median region of thorax with an orange macula; posterolateral angles of pronotum slightly produced	*Chinavia aseada* (Rolston, 1983)
4	Thoracic maculae yellow	5
–	Thoracic maculae orange	6
5	Maculae on the abdominal segments white	*Chinavia impicticornis* (Stål, 1872)
–	Maculae on the abdominal segments yellow and white	*Chinavia nigridorsata* (Breddin, 1901)
6	Maculae on the abdominal segments white	*Chinavia armigera* (Stål, 1859)
–	Maculae on the abdominal segments in other colors	7
7	Median abdominal plates without maculae adjacent to its margins	8
–	Median abdominal plates with maculae adjacent to its margins	9
8	Orange thoracic maculae on pronotal and mesonotal margins	*Chinavia longicorialis* (Breddin, 1901)
–	Orange thoracic maculae on pronotal margins only	*brasicola* (Rolston, 1983)
9	White maculae between median plates	10
–	White maculae laterad to median plates	11
10	Abdomen with one pair (1+1) of yellowish maculae and four pairs (4+4) of white maculae between lateral and median plates	*Chinavia ubica* (Rolston, 1983)
–	Abdomen with one pair (1+1) of yellowish maculae and one pair (1+1) of white maculae near the lateral plates in segments IV and V	*Chinavia obstinata* (Stål, 1860)
11	Abdomen with white maculae; lateral plates with orange maculae in the center	*Chinavia pengue* (Rolston, 1983)
–	Abdomen with white and orange maculae; lateral plates black or with orange maculae in the center	*Chinavia runaspis* (Dallas, 1851)

##### Key to identification of fourth instars of *Chinavia* in Rio Grande do Sul.

**Table:** 

1	Body color predominantly brown to black	2
–	Body color predominantly green and variegated	*Chinavia musiva* (Berg, 1878)
2	Mandibular plates, antennal segment I, apices of femora and bases of tibiae red	*Chinavia erythrocnemis* (Berg, 1878)
–	Legs predominantly black to brown	3
3	Maculae on pronotal and mesonotal margins yellow	4
–	Maculae on pronotal and mesonotal margins orange	5
4	Pronotum trapezoidal; posterolateral angles produced	*Chinavia nigridorsata* (Breddin, 1901)
–	Pronotum trapezoidal; posterolateral angles not produced	*Chinavia impicticornis* (Stål, 1872)
5	Median region of thorax and median abdominal plates with shapeless color maculae	6
–	Median region of thorax and median abdominal plates without color maculae	7
6	Shapeless maculae straw-yellow	*Chinavia armigera* (Stål, 1859)
–	Shapeless maculae orange	*Chinavia aseada* (Rolston, 1983)
7	Abdomen with white maculae between lateral and median abdominal plates	*Chinavia pengue* (Rolston, 1983)
–	Abdomen with white and yellow or orange maculae between lateral and median abdominal plates	8
8	Abdomen with white and orange maculae	9
–	Abdomen with white and yellow maculae	10
9	White maculae on abdomen rounded and similar in size; one pair (1+1) of white maculae laterad to second and third median plates	*Chinavia runaspis* (Dallas, 1851)
–	White maculae on abdomen of different sizes; median plates without laterad maculae	*Chinavia brasicola* (Rolston, 1983)
10	Mesonotum without maculae along the margins; one pair (1+1) of white maculae adjacent to first lateral plates	*Chinavia obstinata* (Stål, 1860)
–	Mesonotum with maculae along lateral margins	11
11	Mandibular plates with reddish orange bands; mesonotum with median maculae; white maculae along posterior margins of median plates	*Chinavia ubica* (Rolston, 1983)
–	Mandibular plates without bands; mesonotum without median maculae; median plates without maculae	*Chinavia longicorialis* (Breddin, 1901)

##### Key to identification of fifth instars of *Chinavia* in Rio Grande do Sul.

**Table:** 

1	Body color predominantly brown to black	2
–	Body color predominantly green and variegated	*Chinavia musiva* (Berg, 1878)
2	Mandibular plates, antennal segment I, apices of femora and bases of tibiae red	*Chinavia erythrocnemis* (Berg, 1878)
–	Legs predominantly black to brown	3
3	Pronotum trapezoidal; posterolateral angles produced	4
–	Pronotum trapezoidal; posterolateral angles not produced	6
4	Thorax variegated, predominantly straw-yellow	*Chinavia armigera* (Stål, 1859)
–	Thorax variegated, predominantly light brown, dark brown or black	5
5	Macula anteriad of the first median plate wider than diameter of eye; light brown band covering almost the entire surface of clypeus; orange maculae covering almost entirely the median plates	*Chinavia aseada* (Rolston, 1983)
–	Macula anteriad of the first median plate smaller than diameter of eye; clypeus immaculate; orange maculae covering almost half of the median plates	*Chinavia nigridorsata* (Breddin, 1901)
6	Lateral plates yellow emarginated in black	*Chinavia impicticornis* (Stål, 1872)
–	Lateral plates orange to red emarginated in black	7
7	Scutellum without median macula	8
–	Scutellum with median macula	9
8	Median plates orange at the middle; abdomen with three pairs of white maculae, between lateral and median plates	*Chinavia pengue* (Rolston, 1983)
–	Median plates green at the middle; abdomen with four pairs of white maculae, between lateral and median plates	*Chinavia longicorialis* (Breddin, 1901)
9	Abdominal maculae white; lateral and median plates with red orange maculae	*Chinavia ubica* (Rolston, 1983)
–	Abdominal maculae white and orange; lateral and median plates with orange maculae	10
10	Mesonotum with small orange maculae, uniformly distributed	*Chinavia runaspis* (Dallas, 1851)
–	Mesonotum with maculae restricted to the median region and margins	11
11	Scutellum at middle with a round macula anteriad of an oblong macula, both orange; abdomen with a pair (1+1) of orange maculae and four pairs (4+4) of small white maculae between lateral and median plates	*Chinavia obstinata* (Stål, 1860)
–	Scutellum at middle with a large orange macula; abdomen with a pair (1+1) of orange maculae and four pairs (4+4) of large white maculae between lateral and median plates	*Chinavia brasicola* (Rolston, 1983)

## Discussion

Recognition of *Chinavia* species at the nymphal stage is based on the general color pattern of the body and number, size and coloration of dorsal maculae (Grazia et al. 1982; Schwertner et al. 2002; Matesco et al. 2003). The first instar of *Chinavia armigera*, *Chinavia aseada*, *Chinavia brasicola* and *Chinavia runaspis* follow the pattern observed for other *Chinavia* species with described immatures: body coloration predominantly dark; head and thorax with a dorsal median yellow to reddish macula; abdomen with a series of white to orange maculae lateral to the first three median plates (Schwertner et al. 2002, Matesco et al. 2009a). *Chinavia musiva* differs from other species by having the general color of abdomen predominantly green and variegated, without maculae (Matesco et al. 2008). The four species studied here have the dorsal median macula orange colored; in *Chinavia armigera* this macula is darker than in the other species. The shape and size of dorsal median macula may also vary. In *Chinavia runaspis*, the shape is round and it is the largest size observed in the species studied so far. Furthermore, of all the *Chinavia* species found in the state of Rio Grande do Sul, only *Chinavia obstinata* has white maculae surrounding all abdominal lateral plates, and the maculae lateral to first median plate are yellowish (Matesco et al. 2003). The remaining species have lateral plates entirely black, without surrounding white maculae. Furthermore, the first instar nymphs of the four species described here have only white maculae on the dorsum of the abdomen.

From the second instar on, nymphs of *Chinavia* have a pair (1+1) of maculae on lateral margins of pronotum, which differ in color as compared to other species: yellow in *Chinavia impicticornis* and *Chinavia nigridorsata*; reddish-orange in *Chinavia pengue* and *Chinavia ubica*, and orange in the remaining species, including those described here. On the abdomen, the anterior median white maculae, the presence of 1+1 yellow or orange maculae lateral to first median plate, and 1+1 white maculae surrounding the fore lateral plates are common features of *Chinavia* nymphs in general (Matesco et al. 2009a). However, *Chinavia armigera* and *Chinavia aseada* are distinguished by not having lateral plates surrounded by maculae and have the abdominal maculae exclusively white. The same can be observed in *Chinavia pengue*, but this species shows areas of head and legs in red as diagnostic characters, besides not having maculae on lateral margins of pronotum.

In the third instar, all the dorsal maculae expand and other maculae with the same color can appear on the mandibular plates and lateral margins of mesonotum, which allow an easier identification of the species. Species that do not have maculae on the mandibular plates and margins of mesonotum are *Chinavia brasicola* and *Chinavia obstinata*.These two species differ from each other in the size of the white maculae located before the first median plate and abdominal maculae, which are broader in these two species. Bands on the mandibular plates, maculae in the middle of the thorax and in the center of median and lateral plates dorsally can appear from the fourth instar on.In *Chinavia armigera*, *Chinavia erythrocnemis* and *Chinavia pengue*, bands on mandibular plates are pale straw-yellow, red and red-orange, respectively. In the fifth instar, the diagnostic characteristics become more evident, allowing easier identifications at the species level. Wing pads surpass or at least reach the posterior margin of the metanotum, and nymphs already exhibit sexual dimorphism: sternum VIII is entire in males and divided into two lobes in females (Dupuis 1947, Brailovskyet al. 1992).

The nymphs of *Chinavia brasicola* are similar to those of *Chinavia runaspis*,as they display a general color predominantly black, and white and orange maculae distributed on the dorsum of the thorax and abdomen. However, the white maculae observed in *Chinavia brasicola* are larger than those observed in *Chinavia runaspis*. In this instar, nymphs of *Chinavia armigera*, *Chinavia aseada* and *Chinavia nigridorsata* are very similar in color pattern, and posterolateral angles of the pronotum are more acute than in the other species. However, the predominant color of the thorax, which is dark brown in *Chinavia nigridorsata*, while predominantly light brown in the two other species may distinguish them. However, some individuals of *Chinavia aseada* and *Chinavia nigridorsata* may have thoracic coloration reddish instead of dark brown. Median dorsal plates of *Chinavia aseada* have orange maculae and *Chinavia nigridorsata* red-orange. Lateral plates of *Chinavia armigera* and *Chinavia nigridorsata* are predominantly orange to orange-red, outlined in black, as in *Chinavia aseada*. However, the black margins of the lateral plates observed in *Chinavia aseada* are narrower than in the two other species mentioned above. The number and distribution of trichobothria in the nymphs of *Chinavia armigera*, *Chinavia aseada*, *Chinavia brasicola* and *Chinavia runaspis* follow the pattern described by Schaefer (1975) for Pentatomoidea, which also has been previously observed in other species of *Chinavia* (Grazia et al. 1982, Matesco et al. 2003, 2007, 2009a).

## Supplementary Material

XML Treatment for Chinavia armigera

XML Treatment for Chinavia aseada

XML Treatment for Chinavia brasicola

XML Treatment for Chinavia runaspis
